# Identification of Rickettsial Infections by Using Cutaneous Swab Specimens and PCR

**DOI:** 10.3201/eid1701.100854

**Published:** 2011-01

**Authors:** Yassina Bechah, Cristina Socolovschi, Didier Raoult

**Affiliations:** Author affiliation: Université de la Méditerranée, Marseille, France

**Keywords:** Rickettsia, Orientia tsutsugamushi, eschars, swabs, PCR, guinea pigs, humans, France, dispatch

## Abstract

To determine the usefulness of noninvasive cutaneous swab specimens for detecting rickettsiae, we tested skin eschars from 6 guinea pigs and from 9 humans. Specimens from eschars in guinea pigs were positive for rickettsiae as long as lesions were present. Optimal storage temperature for specimens was 4°C for 3 days.

Rickettsiae are a group of obligate, intracellular, gram-negative bacteria. The family *Rickettsiaceae* includes the genera *Rickettsia* and *Orientia* (*1*). Rickettsiae are transmitted to humans by arthropods (*2*) and cause diseases characterized by fever, headache, rash, and vasculitis (*3*). An infection eschar is commonly found at the site of the arthropod bite because of local multiplication of the bacteria. Incidence of infection with rickettsiae is increasing worldwide (*4*) in certain disease-endemic foci, and seasonal, sporadic (*5*,*6*), and occasionally epidemic forms have been reported (*7*). Over the past 20 years, advances in molecular techniques and cell culture have facilitated identification of *Rickettsiales*, and new species and diseases have been described (*4*,*8*). Recently, a new *Rickettsia* species, 364D, was identified in patients from California (*9*).

Eschar biopsies are used for detection of *Rickettsia* spp., but this technique is invasive and painful for patients and is difficult to perform for certain areas of the body. Successful diagnosis in patients by using rapid, noninvasive, and painless techniques is beneficial. One study reported the usefulness of swabs of skin lesions in the diagnosis of 3 cases of Queensland tick typhus and 1 case of African tick bite fever (*10*). In addition, eschar crust samples were useful in the diagnosis of 1 case of infection with *Orientia tsutsugamushi*, the infectious agent of scrub typhus (*11*). To evaluate the potential usefulness of swabs of skin lesions for rickettsial diagnosis, we evaluated this procedure for eschars from 6 guinea pigs and 9 patients.

## The Study

The animal study was conducted beginning in February 2009, and the human study was conducted beginning in June 2009. *R. conorii, R. akari, R. rhipicephali*, *R. africae*, *R. parkeri*, and *O. tsutsugamushi* were grown in L929 cell monolayers, purified, and titrated as reported (*12*). A suspension of each rickettsial species (200 μL containing 1 × 10^5^ rickettsia) was injected intradermally into 8 shaved areas on the backs of 6 Hartley guinea pigs (1 species/guinea pig) by using aseptic procedures (*12*). A negative control guinea pig was infected with 200 μL (1 × 10^6^ cells/mL) of an L929 cell suspension. Infection sites were inspected daily for skin lesions. Animals were handled according to the regulations of Décret No. 887–848 du 10/19/1987, Paris. The experimental protocol was reviewed and approved by the Institutional Animal Care Committee, Université de la Méditerranée, Marseille.

Infection with each rickettsial species caused an eschar at the infection site (*12*). Eschars were observed at day 3 postinfection. A sterile cotton swab (Copan Italia S. p. A., Brescia, Italy) was rotated against the eschar (3 circular motions) and stored at 4°C for 24 h. Swabs were then placed in 400 μL of phosphate-buffered saline, and DNA was extracted by using the QIAamp DNA Mini Kit (QIAGEN, Hilden, Germany). Lesions were swabbed daily until the animal showed clinical recovery (day 20 postinfection for those infected with *R. akari*, *R. conorii*, and *R. rhipicephali* and day 13 postinfection for those infected with *R. africae*, *R. parkeri*, and *O. tsutsugamushi*).

**Figure 1:**
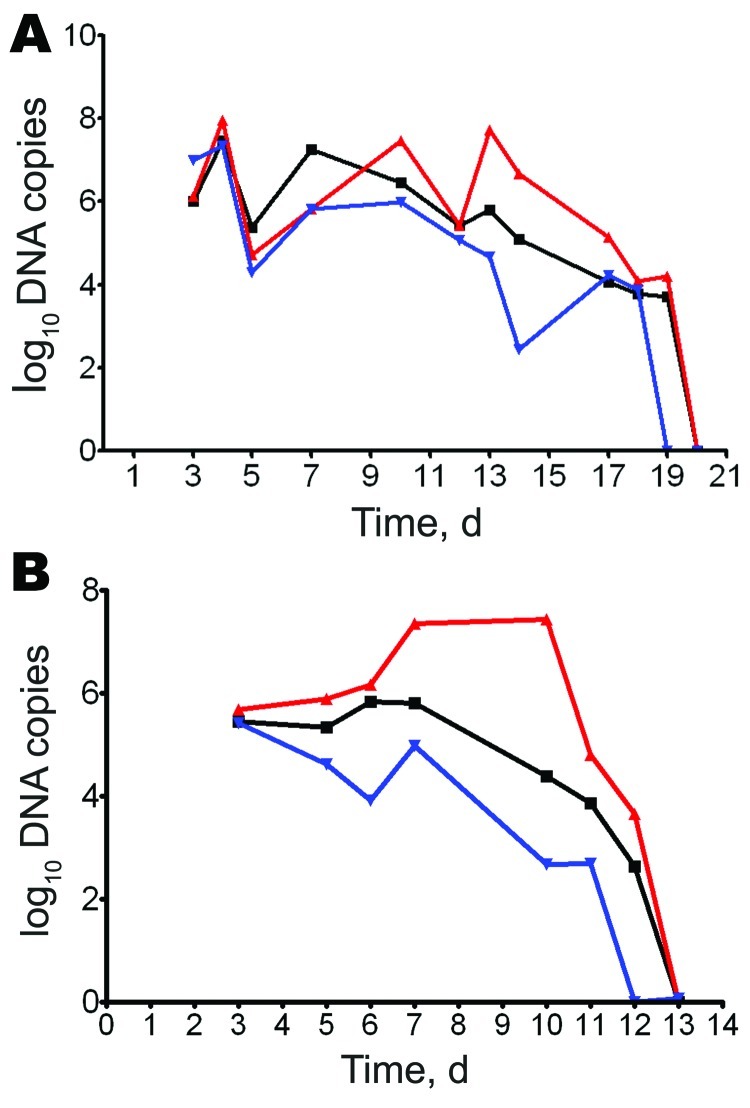
Molecular detection of *Rickettsia* spp. in swabs of skin lesions, Marseille, France. Guinea pigs were infected intradermally with different *Rickettsia* spp., and skin eschar swab specimens were obtained when lesions appeared. Samples (2 ± 1 mg) were tested, and DNA was extracted in a final volume of 100 μL. Number of rickettsial DNA copies was determined by quantitative PCR until day 20 postinfection for *R. akari* (black line), *R. conorii* (red line), and *R. rhipicephali* (blue line) (A) and until day 13 postinfection for *R. africae* (black line), *Orientia tsutsugamushi* (red line), and *R. parkeri* (blue line) (B). Values are copies of citrate synthase A gene*/*5 μL swab extract.

Maximum number of DNA copies for *R. rhipicephali, R. akari*, and *R. conorii* was detected on day 4 postinfection (2.27 × 10^7^, 2.96 × 10^7^, and 9.28 × 10^7^copies/5 μL of swab DNA extracts, respectively (Figure 1, panel A). Maximum number of DNA copies for *R. parkeri* was detected on day 3 postinfection (2.66 × 10^5^ copies/5 μL), for *R. africae* on day 6 postinfection (6.73 × 10^5^ copies/5 μL), and for *O. tsutsugamushi* on day 10 postinfection (2.7 × 10^7^ copies/5 μL) (Figure 1, panel B).

**Figure 2:**
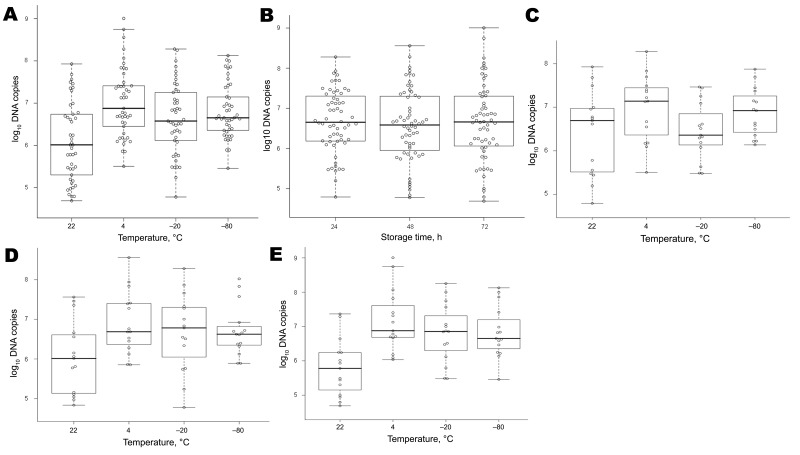
Effect of temperature (A), storage time (B), and temperature and storage times (C–E) on yield of rickettsial DNA, Marseille, France. Guinea pigs (n = 3) were infected with *Rickettsia conorii* and inspected daily for skin lesions. After lesions appeared, 12 swab specimens/animal were obtained daily for 5 days and stored in groups of 3 at 22°C, 4°C, −20°C, or −80°C. DNA was extracted after storage for 24 h, 48 h, or 72 h at each temperature in a final volume of 100 μL, and numbers of bacterial DNA copies were quantified in 5 μL of swab DNA extracts by using quantitative PCR. Box plots indicate 25th and 75th percentiles, horizontal lines indicate medians, and error bars indicate minimum and maximum values.

Effects of temperature and storage time of cotton swabs on bacterial DNA were evaluated in 3 guinea pigs infected with *R. conorii.* Twelve swabs per animal were obtained daily for 5 days and stored in groups of 3 at 22°C, 4°C, −20°C, or −80°C. DNA was extracted after 1, 2, or 3 days of storage. Eschars appeared by day 3 postinfection and reached their maximum size by day 7. Storage at 4°C was the optimal temperature condition for isolation of DNA (7.53 × 10^6^ copies/5 μL vs. 1.03 × 10^6^, 3.77 × 10^6^, or 4.49 × 10^6^ copies/5 μL for swab storage at 22°C, −20°C, or −80°C respectively; p = 0.0001) (Figure 2, panel A). Storage time (24 h, 48 h, and 72 h) had no effect on DNA yield (Figure 2, panel B). Temperature had a significant effect (p<0.05) on DNA yield and for the same extraction (Figure 2, panels C–E).

To demonstrate the usefulness of skin lesion swabs for detection of rickettsial infection, we used this technique with eschars from patients with suspected rickettsioses. Nine patients were included in this experiment after informed consent was obtained. This experiment was reviewed and approved by the local ethics committee (reference 09–016). DNA was extracted from swabs or skin biopsy specimens and tested by quantitative PCRs (qPCRs) (*13*) specific for a fragment of the citrate synthase A gene, which is conserved among spotted fever group rickettsiae, or the gene coding periplasmic serine protease of *O. tsutsugamushi*; β-actin gene was used as a control (*14*).

When rickettsial DNA was amplified in samples, specific qPCR was performed by using specific primers and probes and on the basis of clinical and epidemiologic data (Technical Appendix Table 1 ) (*4*). If specific rickettsial DNA was not detected, PCR amplification and sequencing were performed to identify the causative agent (*4*,*15*). *R*. *montanensis* DNA was used as a positive control, and DNA from sterile biopsy samples and sterile water were used as a negative control.

The qPCR for the β-actin gene showed cycle threshold (C_t_) values of 19–23 for skin biopsy samples and 22–37 for swab samples (Table). Spotted fever group rickettsial DNA was detected in biopsy samples from 5/5 patients and swab samples from 8/9 patients (Technical Appendix Table 2). Specific qPCR showed a diagnostic result in 3/7 swabs samples and 4/5 skin biopsy samples.

**Table:** Table. Molecular results for 9 patients with rickettsioses for identification of *Rickettsia* spp., Marseille, France*

Patient no.	Swab no.	Skin swab specimens, C_t_				Biopsy specimens, C_t_			Final diagnosis
		Actin	Conserved sequence†	Specific sequence‡		Actin	Conserved sequence†	Specific sequence‡	
1	1	22.27	33.91	36.63		19.39	29.03	32.52	*R. conorii*
2	1	29.73	Neg	Neg		21.21	29.32	33.29	*R. conorii*
3	1	22.32	30.99	Neg		18.89	28.72	Neg	*R. sibirica mongolitimonae*
4	1	24.48	35.21	34.15		22.92	31.92	26.66	*R. africae*
5	1	31.13	34.67	Neg		20.68	33.66	31.9	*R. africae*
6§	1	35.49	35.29	Neg		–	–	–	*R. slovaca*
7§	1	24.78	30.63	ND		–	–	–	*R. sibirica mongolitimonae*
8§	1	24.19	Neg	Neg		–	–	–	*R. conorii*
	1	23.36	Neg	Neg		–	–	–	–
	1	21.94	37.97	Neg		–	–	–	–
	1	35.50	Neg	Neg		–	–	–	–
9§¶	1	32.50	Neg	Neg		–	–	–	*R. australis*
	1	32.05	Neg	Neg		–	–	–	*–*
	1	30.95	Pos	Neg		–	–	–	–
	1	24.99	Neg	Neg		–	–	–	–
	1	29.21	Neg	Neg		–	–	–	–
	1	31.78	Neg	Neg		–	–	–	–
	1	35.83	Neg	Neg		–	–	–	–
	1	24.98	Pos	Pos		–	–	–	–
	1	35.5	Neg	Pos		–	–	–	–
	1	36.96	Neg	Neg		–	–	–	–
	1	32.21	Neg	Neg		–	–	–	–

*C_t_, cycle threshold; neg, negative; –, not applicable; ND, not done; pos, positive. †Rickettsial DNA was identified by using a fragment of the citrate synthase A gene that is conserved among all spotted fever group rickettsiae. ‡Specific quantitative PCR was performed on the basis of epidemiologic data and tick bite history of each patient. §No cutaneous biopsy samples were available. ¶Results for patient 9 correspond to results of re-amplification of products of the first PCRs.

We amplified *R. conorii* DNA from patients 1 and 2, *R. africae* DNA from patients 4 and 5, and *R. australis* DNA from patient 9. Rickettsial DNA from patients 3 and 7 showed 100% homology with the *R. sibirica mongolitimonae* citrate synthase A gene (GenBank accession nos. DQ097081 and DQ423370, respectively). Rickettsial DNA from patient 6 showed 99.1% homology with DNA from *R. slovaca*. Patient 9 was a technician who was accidentally infected by the aerosol route when handling *R. australis*. Only 2/11 swabs obtained from vesicular lesions of patient 9 were positive for rickettsial DNA and *R. australis* DNA after reamplification of primary PCR products. These samples showed 98% homology with *R. australis* 23S rRNA gene (GenBank accession no. AJ133711) (Technical Appendix Table 2).

## Conclusions

Our study showed the efficacy and reliability of skin lesion swabs for molecular detection of 6 *Rickettsia* species (Figure 1). Rickettsial DNAs were detected by using this technique as long as eschars persisted (<19 days). For short-term storage of swabs, 4°C was the optimal temperature. Using swabs of eschars, we made a diagnosis of rickettsiosis for 8/9 patients. For patients 6, 7, and 8, for whom biopsy samples were not available, we confirmed the diagnosis by using swab samples. We also showed that for patient 9, who had a rickettsiosis but no eschar, swabbing of vesicular lesions may be useful for diagnosis, although these lesions were less sensitive than eschars.

Our results indicate that swabs of eschars can be used for molecular detection of rickettsial infections when biopsy samples are not available or biopsies are difficult to perform. We recommend that swabs be used for DNA extraction immediately after sampling or stored at 4°C until needed.

## Supplementary Material

Appendix Technical Appendix.
