# Supplementary material for: Identification of Rickettsial Infections by Using Cutaneous Swab Specimens and PCR
Source: Emerg Infect Dis. 2011 Jan;17(1):83–6. doi: 10.3201/eid1701.100854 (PMC3375762; doi:10.3201/eid1701.100854)
Supplement: Appendix — Technical Appendix. [file 10-0854-app.pdf]

Article DOI: 10.3201/eid1701.100854

Identification of Rickettsial Infections by Using Cutaneous Swab Specimens and PCR

Technical Appendix

Technical Appendix Table 1. Nucleotide sequences of primers and probes used for identification of *Rickettsia* spp., Marseille, France*

Gene	Organism	Primer, 5′ → 3′	Primer, 3′ → 5′	Probe, 5′ → 3′
*glt*A	SFG rickettsiae	GTG AAT GAA AGA TTA CAC TAT TTAT	GTA TCT TAG CAA TCA TTC TAA TAG C	FAM-CTA TTA TGC TTG CGG CTG TCG GTT C-TAMRA
Putative acetyltransferase	*Rickettsia conorii conorii*	TTG GTA GGC AAG TAG CTA AGC AAA	GGA AGT ATA TGG GAA TGC TTT GAA	FAM-GCG GTT ATT CCT GAA AAT AAG CCG GCA-TAMRA
ITS	*R. africae*	TGC AAC ACG AAG CAC AAA AC	CCT CTT GCG AAA CTC TAC TTT TGA	6-FAM-CGT GTG GAT TCG AGC ACC GGA-TAMRA
Intergenic spacer	*R. slovaca*	GCA ACG GTT TTT GGT ATC GT	AAT CGA ATG CAC CAC CAC TT	6-FAM-TCC CGT CCC AGC CAT TCG TC-TAMRA
Not specified	*R. raoultii*	CCA ATA CCT TGC CCA AAA CA	AAA TTG ATG GTG CAG GAG TGC	FAM-TGG GGC TTT TTC ATG TCC TAA GCA CA-TAMRA
23S rRNA	*R. australis*	TGC ACA TTA AGT GCG AGT GG	GGA CTT TCG GCG AGA ATG TT	FAM-TCC GTA AGC CTG TGA AGG TGA ACC G-TAMRA
Periplasmic serine protease	*Orientia tsutsugamushi*	AAC TGA TTT TAT TCA AAC TAA TGC T	TAT GCC TGA GTA AGA TAC RTG AAT RGA ATT	FAM-TGG GTA GCT TTG GTG GAC CGA TGT TTA ATC T-TAMRA

**glt*A*, citrate synthase; SFG, spotted fever group; ITS, internal transcribed spacer.

Technical Appendix Table 2. Clinical features of 9 patients with rickettsioses and results of molecular assays for identification of *Rickettsia* spp, Marseille, France*

		Clinical manifestations			Molecular diagnostic results			
Patient no.	Travel destination	Eschar location (no.)	Rash	Lymphadenopathy	Conserved sequence†	Specific sequence‡	Sequencing	Final diagnosis
1§	Algeria	Inguinal (1)	Maculopapular	No	Pos (1/1)	*R. conorii* (1/1)	ND	*R. conorii* infection (Mediterranean spotted fever)
2§	Southern France	Cervical (1)	Maculopapular	No	Neg (1/1)	Neg (1/1)	ND	*R. conorii* infection (Mediterranean spotted fever)
3§	Egypt	Scapula (1)	Maculopapular	Axillar	Pos (1/1)	ND	*R. sibirica mongolitimonae*	*R. sibirica mongolitimonae* infection (lymphangitis-associated rickettsiosis)
4§	South Africa	Foot/buttocks (2)	Vesicular	Inguinal	Pos (1/1)	*R. africae* (1/1)	ND	*R. africae* infection (African tick bite fever)
5§	South Africa	Buttocks/leg (2)	No	Inguinal	Pos (1/1)	Neg (1/1)	ND	*R. africae* infection (African tick bite fever)
6	France	Scalp (1)	No	Cervical	Pos (1/1)	Neg (1/1)	*R. slovaca*	*R. slovaca* infection (scalp eschar and neck lymphadenopathy)
7	France	Leg (1)	Maculopapular	Inguinal	Pos (1/1)	ND	*R. sibirica mongolitimonae*	*R. sibirica mongolitimonae* infection (lymphangitis-associated rickettsiosis)
8	France	Leg (1)	Maculopapular	No	Pos (1/3)	Neg (3/3)	Negative	*R. conorii* infection (Mediterranean spotted fever)
9	France	No	Macular and vesicular	No	Pos (2/11)	*R. australis* (2/11)	*R. australis*	*R. australis* infection (Queensland tick typhus)

*All 9 patients had fever. Pos, positive; ND, not done; Neg, negative. Values in parentheses indicate no. eschars or no. positive swabs/no. of sampled swabs.
†Rickettsial DNA was identified by using a fragment of the citrate synthase A gene, which is conserved among all spotted fever group rickettsiae.
‡Specific quantitative PCR was performed on the basis of epidemiologic data and tick bite history of each patient.
§Eschar biopsies were performed and showed positive results.
